# In Vitro Skin Penetration of 5α-Reductase Inhibitors from *Tectona grandis* L.f. Leaf Extracts

**DOI:** 10.3390/molecules30051151

**Published:** 2025-03-04

**Authors:** Kamonlak Insumrong, Neti Waranuch, Kornkanok Ingkaninan, Nutchaninad Tanuphol, Abhay Prakash Mishra, Wudtichai Wisuitiprot, Eakkaluk Wongwad, Ngamrayu Ngamdokmai, Nungruthai Suphrom

**Affiliations:** 1 Department of Chemistry, Faculty of Science, Naresuan University, Phitsanulok 65000, Thailand; insumrong.k@gmail.com; 2 Department of Pharmaceutical Technology, Faculty of Pharmaceutical Sciences and Center of Excellence for Innovation in Chemistry, Naresuan University, Phitsanulok 65000, Thailand; 3 Cosmetics and Natural Products Research Center, Faculty of Pharmaceutical Sciences, Naresuan University, Phitsanulok 65000, Thailand; abhaypharmachemhnbgu@gmail.com; 4 Center of Excellence for Natural Health Product Innovation and Center of Excellence for Innovation in Chemistry, Department of Pharmaceutical Chemistry and Pharmacognosy, Faculty of Pharmaceutical Sciences, Naresuan University, Phitsanulok 65000, Thailand; k_ingkaninan@yahoo.com (K.I.); nutchaninad789@gmail.com (N.T.); 5 Sirindhorn College of Public Health Phisanulok, Faculty of Public Health and Allied Health Sciences, Praboromarajchanok Institute, Ministry of Public Health, Phitsanulok 65130, Thailand; wisuitiprot@hotmail.com; 6 Department of Cosmetic Sciences, School of Pharmaceutical Sciences, University of Phayao, Phayao 56000, Thailand; e.wongwad@yahoo.com; 7 Department of Applied Thai Traditional Medicine, School of Medicine, Walailak University, Thasala, Nakhon Si Thammarat 80160, Thailand; ngamrayu.ng@mail.wu.ac.th; 8 Department of Chemistry, Faculty of Science and Center of Excellence for Innovation in Chemistry, Naresuan University, Phitsanulok 65000, Thailand

**Keywords:** *Tectona grandis*, 5α-reductase inhibitors, penetration, partition coefficient, chemical analysis

## Abstract

The leaf extract of *Tectona grandis* L.f. has shown potential as a 5α-reductase inhibitor, with two bioactive markers, namely (+)-eperua-8,13-dien-15-oic acid (**1**) and (+)-eperua-7,13-dien-15-oic acid (**2**), used for extract standardization. The purpose of this research was to investigate the in vitro skin penetration behavior of **1** and **2** in *T. grandis* leaf ethanolic extract solution and ready-to-use extract in propylene glycol (PG), and secondly, to determine their physicochemical properties, including partition coefficients and solubility. The appropriate vehicle for the in vitro skin penetration study was evaluated using the shake-flask method. The in vitro skin penetration study was conducted using the Franz diffusion cell model, and the amounts of the two active compounds in the extracts were analyzed using the HPLC method. Compounds **1** and **2** showed poor solubility in distilled water, whereas their solubility in HEPES buffer with 2% *w*/*v* of Tween 20 was significantly greater. The partition coefficient (log P_o/w_) value for **1** was 5.77 ± 0.07, and for **2**, it was 5.66 ± 0.02, indicating that both compounds are hydrophobic. After 24 h of an in vitro skin penetration study, **1** in both extracts showed significantly higher cumulative amounts (%) compared to **2**. These findings suggest that **1** is more hydrophobic and readily penetrates the stratum corneum. When a PG enhancer was added, high cumulative amount trends of **1** and **2** in the ethanolic extract and extract in PG in the receiver compartment were detected after 24 h. These studies provide important insights that will guide the further development of products with *T. grandis* extracts for treating hair loss.

## 1. Introduction

*Tectona grandis* L.f. is a commercially important and widely cultivated industrial crop. This plant is known as the teak tree (English) or sak (Thai). There are only three species in the genus *Tectona*, namely *T. grandis*, *Tectona hamiltoniana* Wall., and *Tectona philippinensis* Benth. & Hook.f., which belong to the family Lamiaceae. *T. grandis* is a large deciduous tree that grows to heights of 30–40 m and is found mostly in Southeast Asia [1,2]. Teak wood is used in the timber industry due to its good texture, color, and finishing qualities [1].

**Figure 1:**
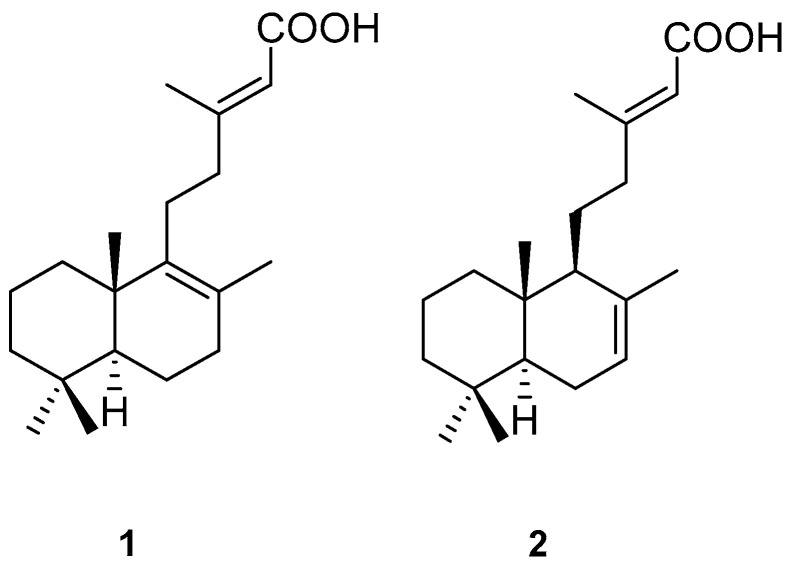
Chemical structures of 5α-reductase inhibitors from *T. grandis* leaf extract.

Several parts of *T. grandis* have traditionally been used for the treatment of a wide range of conditions, including bronchitis, inflammation, skin diseases, stomatitis, and urinary retention [2,3]. In Indian traditional medicine, the seeds have been recommended as a hair growth promoter [3]. Additionally, *T. grandis* possesses a wide range of pharmacological actions, including antibacterial [4], cytotoxic [5,6], antioxidant [7], hypoglycemic [8], anti-inflammatory [9,10], phytotoxic [11], antiplasmodial [12], and antipyretic [13] properties, and wound-healing [14] activitiy. An in vivo investigation of petroleum ether extract of *T. grandis* seeds in the albino mice model revealed that treatment with 5% and 10% petroleum ether extract increased the number of hair follicles more effectively than a standard positive drug, minoxidil [15]. In addition, a study by Fachrunniza et al. [16] described the biological activities of *T. grandis* related to hair loss treatment, which included anti-testosterone and anti-inflammatory activities through interleukin 1 beta (IL-1β) secretion suppression. Among the extracts from the various parts of *T. grandis*, the hexane and ethyl acetate leaf extracts exhibited potent 5α-reductase inhibitory activity. The cytotoxic effect of the hexane extract on human follicle dermal papilla cells (HFDPCs) was lower than that of the ethyl acetate extract. These findings showed that the *T. grandis* leaf extract might serve as a new source of active ingredients for alternative medicines or cosmetics for hair loss treatment. We previously isolated and identified two 5α-reductase inhibitors from *T. grandis* leaf extract: (+)-eperua-8,13-dien-15-oic acid (**1**) and (+)-eperua-7,13-dien-15-oic acid (**2**) (Figure 1). Two bioactive compounds (**1** and **2**) in *T. grandis* leaf extracts were quantified and used as markers for the further development of products [17].

Furthermore, in the food, cosmetic, and pharmaceutical industries, natural crude extracts prepared in carrier solvents are often referred to as ready-to-use. Propylene glycol (PG) is one such carrier solvent, also called an additive solvent, utilized in these sectors to absorb water. The Food and Drug Administration (FDA) has approved PG, which is generally considered safe for use in food. It is employed to retain moisture in pharmaceuticals, cosmetics, and food products [18,19]. Thus, the ready-to-use extract prepared with PG solvent is an appealing choice to investigate the pre-formulation of products for the development of medicine or cosmetic products. The pre-formulation study is essential for ensuring that the final formulation of products is safe to use and maintains the quality of the extract. There is currently no information in the literature on a pre-formulation study of the two 5α-reductase inhibitors (**1** and **2**) in *T. grandis* extract.

Thus, this study aimed to evaluate the in vitro skin penetration behavior of compounds **1** and **2** in *T. grandis* leaf ethanolic extract and ready-to-use extract in PG. Additionally, their physicochemical properties, such as partition coefficients and solubility, were examined.

## 2. Results and Discussion

### 2.1. Partition Coefficients

**Figure 2:**
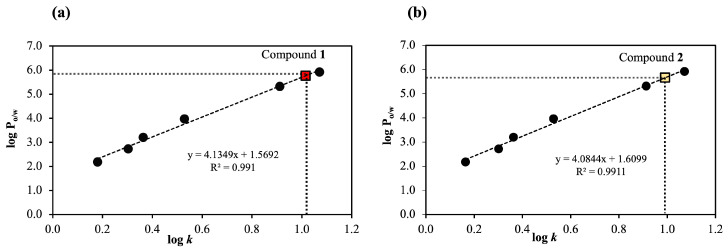
The correlation plotted the log P_(o/w)_ of reference compounds against their average log *k* (*n* = 5). The log *k* and log P_(o/w)_ values of (**a**) compound **1** were 1.015 ± 0.016 and 5.767 ± 0.07, respectively, and those of (**b**) compound **2** were 0.992 ± 0.005 and 5.661 ± 0.02, respectively.

The partition coefficient (log P_(o/w)_) is the diffusion coefficient of compounds when they are dissolved in an immiscible biphasic system between *n*-octanol and water, which describes the hydrophilicity (water liking) and hydrophobicity (lipid liking) of the agent. Therefore, the log P_(o/w)_ value can be used to predict a compound’s capacity to penetrate the stratum corneum (a lipid domain) and the viable epidermis (an aqueous domain) [20]. In our work, the log P_(o/w)_ values of **1** and **2** were determined using HPLC, and the *k* values of the reference and test samples were calculated using their retention times. The log P_(o/w)_ values of **1** and **2** are reported here for the first time. When log P_(o/w)_ is positive or high, it indicates that the retention time is longer, resulting in a greater *k* value. The relationships between log P_(o/w)_ values of reference compounds and their mean log *k* values were plotted to provide the correlation coefficient (R^2^) of 0.9910 for **1** (Figure 2a) and 0.9911 for **2** (Figure 2b). The *k* values for **1** and **2** were 1.015 ± 0.016 and 0.992 ± 0.005, corresponding to the log P_(o/w)_ values of 5.767 ± 0.07 and 5.661 ± 0.02, respectively.

These results demonstrated that **1** and **2** are hydrophobic compounds. Therefore, they may more easily penetrate the lipid layer of the stratum corneum. This is also suggested by previous reports indicating that hydrophobic compounds tend to increase skin penetration into the outer layer of the skin more than hydrophilic compounds [21].

### 2.2. Solubility of ***1*** and ***2*** in T. grandis Ethanolic Extract

The solubility of **1** and **2** in the crude ethanolic extract was investigated using the shake-flask method at 37 ± 2 °C to determine the solubility in water and a suitable vehicle for the in vitro skin penetration study. For the skin penetration test, HEPES buffer with 2% *w*/*v* of Tween 20 was used as a receptor fluid. An excess of ethanolic extract was added to 1 mL of each solvent for solubility analysis using the HPLC method. In comparison, the solubility of **1** and **2** in distilled water was 6.32 ± 0.42 µg/mL for **1** and 7.79 ± 0.05 µg/mL for **2**, whereas the solubility of these compounds in the HEPES buffer with 2% *w*/*v* of Tween 20 was found to be significantly higher, at 1051 ± 44 µg/mL for **1** and 1080 ± 24 µg/mL for **2**. The solubility values of **1** and **2** are shown in Table 1. Following the United States Pharmacopeia (USP) and British Pharmacopeia (BP), the classification of solubility, regardless of the solvent used, is based only on quantitative considerations [22]. Compounds **1** and **2** were defined as practically insoluble compounds when solubilized in distilled water [23]. These findings indicate that the HEPES buffer with 2% *w*/*v* of Tween 20 is a suitable vehicle for further skin penetration studies.

Furthermore, according to this solubility test, adding chemical enhancers to the extract, such as PG, glycerine, and PEG-40 hydrogenated castor oil, might assist components with low solubility in penetrating more deeply beyond the stratum corneum due to their solubility-enhancing property for hydrophobic compounds [24]. For our study, the most preferred chemical enhancers were PG and PEG-40 hydrogenated castor oil, which were dissolved in extracts to improve their solubility and skin absorption.

### 2.3. Skin Penetration of ***1*** and ***2*** from T. grandis Ethanolic Extract and Extract in PG

**Figure 3:**
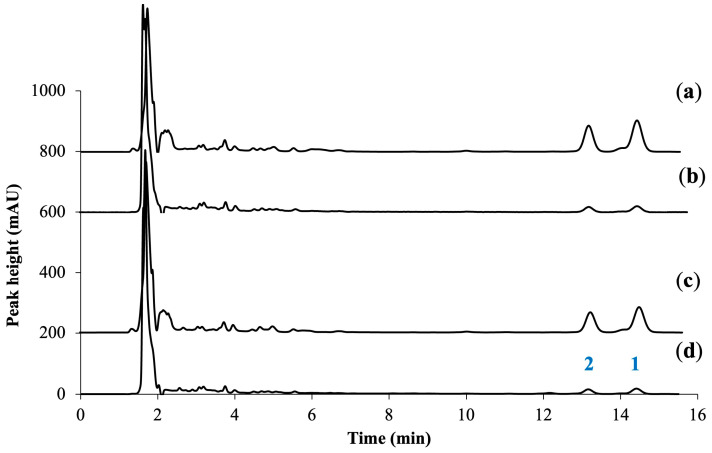
HPLC chromatograms of compounds **1** and **2** in samples: (**a**) ethanolic extract solution before being applied through the membrane, (**b**) ethanolic extract solution after penetration through the membrane at 24 h, (**c**) extract in PG before being applied through the membrane, and (**d**) extract in PG after penetration through the membrane at 24 h. The samples in (**b**,**d**) were collected from the receiver compartment.

Franz diffusion cells were used to assess the skin penetration of **1** and **2** in the ethanolic extract and those of the extract in PG. The amounts of two active compounds in samples were analyzed using HPLC. The HPLC chromatograms before and after penetration (at 24 h) of the ethanolic extract solution and extract in PG through the membrane are shown in Figure 3. The retention times of **1** and **2** were monitored at 14.5 and 13.1 min, respectively. The chromatographic analysis of receiver solutions from the receiver compartment (Figure 3b,d) showed that the two marker compounds in the extracts successfully penetrated the skin membrane, but their peak heights were significantly lower than the initial extract solution (Figure 3a,c), suggesting limited penetration.

**Figure 4:**
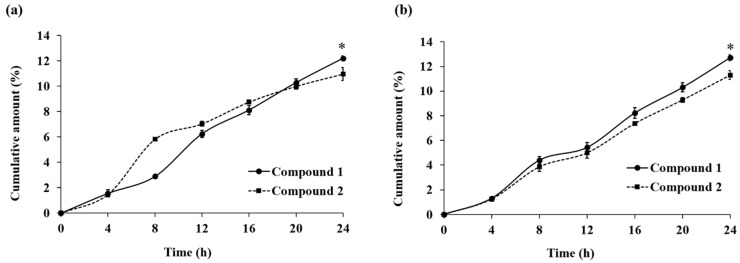
The skin penetration profiles of **1** and **2** after the application of (**a**) ethanolic extract solution and (**b**) extract in PG on the skin membranes for 24 h. Each point represents the cumulative percentage of **1** and **2** in the receptor medium at each time point as measured by the HPLC method. The values represent the means ± SD of triplicate experiments. (* *p* < 0.05, significantly different compared with **2** at 24 h).

As shown in Figure 4, the results showed that **1** and **2** in the ethanolic extract solution were discovered in the receiver compartment after 4 h and exhibited high cumulative amounts (>10%) after 24 h. The cumulative amounts after 24 h were 12.2 ± 0.2% for **1** and 11.0 ± 0.5% for **2** (Figure 4a), whereas the extract in PG showed superior skin penetration with cumulative amounts of 12.7 ± 0.2% for **1** and 11.3 ± 0.4% for **2** (Figure 4b). The range of sample recovery was between 90% and 100% (Table 2). These studies suggest that **1** and **2** penetrate into the receiver compartment more effectively when PG solvent is added. However, the greater penetration of **1** after 24 h in both the ethanolic extract solution and the extract in PG indicated significantly larger cumulative amounts (%) than the penetration of **2**. According to the partition coefficient described above, these results revealed that **1** was more hydrophobic than **2**, thereby tending to facilitate its interaction with the lipid layer part of the stratum corneum [25,26]. Both of these compounds have identical molecular weights but differ in the double bond position between C-7 and C-8. There may be other factors for the differences in the penetration profile, like the shape of molecules, the presence of steric hindrance, or the way the compounds interact with the skin membrane [27,28,29]. Therefore, the result of this study may provide useful information for the development of cosmetics or hair loss treatments containing *T. grandis* extracts in the future.

Several articles have demonstrated that terpenes, particularly essential oils (e.g., 1,8-cineole, menthol, and menthone) and their constituents, can enhance percutaneous absorption [24,30]. The study by Srivilai et al. [31] also reported the effects on minoxidil penetration of germacrone and sesquiterpene-enriched extracts from *Curcuma aeruginosa* Roxb., using Franz diffusion cells. Minoxidil penetration alone through human foreskin was limited, reaching just 0.9 ± 0.1% after 8 h in the stratum corneum but was about 10-fold higher when applied in combination with germacrone, and 2% *C. aeruginosa* essential oil (7–8%), both of which enhance skin penetration.

Few studies have previously been conducted on the skin penetration of diterpenes and triterpenes [32,33]. Rizwan et al. [32] used an automated transdermal diffusion cell sampling technique to examine the effects of several monoterpene enhancers and the labdane diterpene forskolin. Terpene enhancers (1% *w*/*w*) were shown to be effective for penetration of rat skin and human cadaver skin models in the following order: cineole > D-limonene > L-menthol > linalool > forskolin and cineole > D-limonene > linalool > L-menthol > forskolin. Although the diterpene forskolin penetrated less than monoterpenes in both skin models, it enhanced skin permeability by disrupting and extracting stratum corneum lipid bilayers, as did other terpenes. Our findings also revealed that these two diterpenes (**1** and **2**) can penetrate the skin by interacting with the lipid layer part of the stratum corneum. However, notably, our research is the first to report the skin penetration of the two 5α-reductase inhibitors (**1** and **2**) in *T. grandis* leaf extracts.

## 3. Materials and Methods

### 3.1. Chemicals

Analytical-grade 95% ethanol was purchased from CHEMIPAN (Bangkok, Thailand). Formic acid was purchased from KemAus (Cherrybrook, NSW, Australia). Acetonitrile and methanol (HPLC grade) were bought from RCI Labscan Ltd. (Bangkok, Thailand). Butylated hydroxytoluene, benzophenone, and 2-[4-(2-hydroxyethyl)piperazin-1-yl] ethanesulfonic acid (HEPES) were purchased from Sigma-Aldrich (St. Louis, MO, USA). Polyethylene glycol-40 (PEG-40) hydrogenated castor oil was purchased from Phitsanuchemicals (Phitsanulok, Thailand). Tween 20^®^ was purchased from AppliChem GmbH (Darmstadt, Germany). Propylene glycol (PG) was purchased from Ajax Finechem (Taren Point, Australia). Benzene was purchased from Carlo Erba (Milan, Italy). Toluene and octyl methoxycinnamate were purchased from Merck (Darmstadt, Germany). Benzyl benzoate was bought from Thai–China Flavours & Fragrance industry (Phra Nakhon Si Ayutthaya, Thailand). The reference 5α-reductase inhibitors, (+)-eperua-8,13-dien-15-oic acid (**1**) and (+)-eperua-7,13-dien-15-oic acid (**2**) were previously isolated from *T. grandis* leaf. These two compounds were identified based on spectroscopic data using NMR, FTIR, and MS. The purities of **1** and **2** were calculated at 99% by the HPLC method [17].

### 3.2. General Experimental Procedures

In the partition coefficient, solubility, and skin penetration tests, the quantities of **1** and **2** in *T. grandis* extracts were analyzed using high-performance liquid chromatography (HPLC): a Shimadzu model LC-20A (for the partition coefficient test) and an Agilent Technology model 1260 infinity (for skin penetration and solubility tests), equipped with UV/Visible detectors. A Phenomenex Luna C18 column (150 × 4.6 mm, 5 µm particle size) and a guard column (5 µm Phenomenex C18, 4 mm × 3 mm) were used for HPLC analysis. Franz diffusion was performed using Logan Instruments Corp (model, FDC-6, Somerset, NJ, USA). An orbital shaker (Biosan, Latvia) was used for solubility testing.

### 3.3. Plant Material

*T. grandis* leaves were collected from Banna District, Nakhon Nayok Province, Thailand, in September 2019. The plant material was identified by Assist. Prof. Dr. Pranee Nangngam, Faculty of Science, Naresuan University, Phitsanulok, Thailand, and a voucher specimen (collection no. 05721) was kept at the Department of Biology in the Faculty of Science, Naresuan University, Phitsanulok, Thailand.

### 3.4. Preparation of T. grandis Leaf Extracts

The mature, fresh leaves of *T. grandis* were cut into small pieces and dried at 55 °C. The dried material was crushed into a fine powder and passed through a 60-mesh sieve. For the preparation of the crude ethanolic extract, the *T. grandis* leaf powder (292 g) was macerated with 95% ethanol (1.17 L) three times (for at least five days each time) at room temperature with occasional shaking. The filtrates were combined and evaporated under reduced pressure to obtain a dark green viscous crude ethanolic extract (32.90 g, 11.27% yield). For the preparation of the ready-to-use extract in PG, a crude ethanolic extract was solubilized with PEG-40 hydrogenated castor oil at a ratio of 1:3 *w*/*w* to produce an extract solution, which was then mixed with PG at a ratio of 1:4 *w*/*w* to obtain a ready-to-use extract in PG. The crude ethanolic extract and the ready-to-use extract in PG were kept at −20 °C until used.

### 3.5. Determination of Partition Coefficients of Compounds ***1*** and ***2***

The partition coefficients *n*-octanol/water (P_(o/w)_) of **1** and **2** were determined by the HPLC method as described in the OECD test guideline no. 117 [34]. This approach applies to log P_(o/w)_ values between 0 and 6. In brief, the following reference compounds with known log P_(o/w)_ were employed as reference substances: benzene (2.177), toluene (2.720), benzophenone (3.200), benzyl benzoate (3.969), butylated hydroxytoluene (5.319), and octyl methoxycinnamate (5.921). The test reference substances and test compounds (**1** and **2**) were dissolved at a concentration of 500 µg/mL in methanol. HPLC was carried out using a Shimadzu model LC-20A equipped with a column C18 as described above. The following HPLC conditions were used: 20 µL of injection volume, a UV detector set at 254 nm, and a column temperature of 25 °C. The compounds were eluted at 1.0 mL/min using an isocratic mode of 60% (*v*/*v*) acetonitrile in water for 16 min. The capacity factor (*k*) of reference and tested compounds was calculated using Equation (1).

*k* = [(retention time − dead time)/dead time] (1)

The experiment was conducted in five replicates (*n* = 5), and the octanol/water partition coefficient (log P_(o/w)_) of reference compounds was plotted against their average log *k* to provide a correlation curve form. The log P_(o/w)_ values of **1** and **2** were calculated.

### 3.6. Solubility Study of Compounds ***1*** and ***2*** in T. grandis Ethanolic Extract

To determine the ability to be dissolved in water and find the suitable receptor fluid for an in vitro skin penetration study, the solubility of **1** and **2** in the extract was evaluated using distilled water and HEPES buffer at pH 7.4 (containing 25 mM HEPES and 2% *w*/*v* of Tween 20^®^). The shake-flask method was used as described in OECD guideline No. 105 [35]. Briefly, excess quantities of extract were added to 1 mL of each solvent and then shaken at 220 rpm (37 ± 2 °C) using an orbital shaker. This process was performed in triplicate. After 24, 48, and 72 h, the tubes were collected and centrifuged at 600× *g*. The clear solutions of distilled water and HEPES buffer (diluted 100-fold with methanol) were then filtered through a 0.45 µm membrane and analyzed using the HPLC method.

### 3.7. In Vitro Skin Penetration Study

Following OECD test no 428 [36], Franz diffusion cells were used to evaluate the skin penetration of the ethanolic extract and extract in PG. As a model, a synthetic membrane (Strat-M™) was employed. The Franz apparatus comprised a 1.76 cm^2^ donor chamber and a 7 mL receiver compartment. The system was maintained at a temperature of 37 °C using a heated water jacket to ensure that the temperature of the membrane surface was 32 ± 1 °C. The solution in the receiver chamber was continually stirred at 600 rpm with a magnetic bar. The receiver chamber solution was made by combining 2% *w*/*v* of Tween 20^®^ in aqueous HEPES (25 mM) buffer pH 7.4. The synthetic membrane was allowed to equilibrate for 30 min. Test sample solutions were prepared using two methods: (i) the ethanolic extract was dissolved with PEG-40 hydrogenated castor oil in a 1:3 *w*/*w* ratio to produce an extract solution, which was then diluted with water in a 1:4 *w*/*w* ratio to produce the ethanolic extract solution, and (ii) the ethanolic extract was prepared using the same procedure as described above, except that in the final step, water was replaced with PG solvent to provide the extract in PG. In the donor chamber, 100 µL of each tested sample solution was placed in contact with the shiny side of a Strat-M™ membrane. At 4, 8, 12, 16, 20, and 24 h, the receiver solution (500 µL) was sampled and replaced with an equal volume of fresh medium. The amounts of the two active compounds in the samples were analyzed using the HPLC method. After 24 h, the remaining test sample on the membrane surface was lightly brushed with a cotton-tipped swab until it appeared shiny. Each cotton-tipped swab and membrane was extracted by sonication with methanol for 15 min. The quantities of the two active compounds in the samples were then measured using HPLC and the percentages of the compounds remaining in the different compartments, relative to the quantity added to the synthetic membrane, were calculated. The experiment was conducted in triplicate for each sample.

### 3.8. HPLC Analysis of Compounds ***1*** and ***2*** in T. grandis Extract

In the solubility and skin penetration studies, the quantification of the two 5*α*-reductase inhibitors (**1** and **2**) in *T. grandis* extracts was performed using an HPLC-UV detector equipped with a column C18(2) as mentioned above. The previously validated method, from [17], was used to conduct the analysis. Briefly, the isocratic elution system was used at a 0.8 mL/min flow rate with a mixture solution of acetonitrile and 0.1% (*v*/*v*) formic acid in purified water (85:15 *v*/*v*) for 16 min. A UV detector was used for chromatographic detection at 220 nm. The volume of injection was 20 µL. The quantities of the two markers in *T. grandis* extracts were calculated from the calibration curves: y = 63.843x + 40.465 (for **1**) and y = 75.954x + 20.8 (for **2**).

### 3.9. Statistical Analysis

The data were expressed as the mean ± standard deviation (SD) of at least triplicate experiments. One-way analysis of variance (ANOVA) was used to assess statistical comparisons, followed by Duncan’s test. The significance level of *p* < 0.05 was considered significant in all cases.

## 4. Conclusions

Based on the results of the in vitro skin penetration study, both 5α-reductase inhibitors (**1** and **2**) in the ethanolic extract and extract in PG were able to initiate skin penetration after 4 h and exhibited high cumulative amounts >10% after 24 h. Moreover, both compounds tended to penetrate the skin more effectively when the PG solvent was used. As a result, our findings verify the efficacy of **1** and **2** in *T. grandis* extract in PG for cosmetic applications. Furthermore, the studies provide important information that can enhance the development of future formulations for topical applications of hair loss treatments containing *T. grandis* extracts.

## Figures and Tables

**Table 1:** Solubility analysis of **1** and **2** contents in the ethanolic extract against these two solvents. The results are expressed as the means ± standard deviation (SD) of triplicate experiments.

Solvents	Amounts (µg/mL)		Solubility Definition
	1	2	
Distilled water	6.32 ± 0.42	7.79 ± 0.05	practically insoluble
HEPES buffer with 2% *w*/*v* of Tween 20	1051 ± 44 *	1080 ± 24 *	slightly soluble

* *p* < 0.05 indicates significant differences in the concentration of the same compound between the two solvents.

**Table 2:** In vitro skin penetration of **1** and **2** after the application of an ethanolic extract solution and extract in PG on the skin membranes for 24 h. The results are expressed as the mean of the percentage (%) applied dose ± SD of triplicate experiments.

Samples	Compounds	Donor (%)	Membrane (%)	Receiver (%)	Recovery (%)
Ethanolic extract solution	**1**	76.16 ± 0.99	4.47 ± 0.14	12.20 ± 0.19 *	92.63 ± 0.84
	**2**	77.20 ± 0.77	5.70 ± 0.37	10.96 ± 0.51	93.86 ± 0.91
Extract in PG	**1**	82.00 ± 0.63	4.11 ± 0.05	12.72 ± 0.24 *	98.84 ± 0.91
	**2**	78.47 ± 0.55	5.03 ± 0.01	11.30 ± 0.35	94.80 ± 0.85

* *p* < 0.05, significantly different compared with **2** in the same extract.

## Data Availability

The data presented in this study are available upon request from the corresponding authors.
